# State Space Correspondence and Cross-Entropy Methods in the Assessment of Bidirectional Cardiorespiratory Coupling in Heart Failure

**DOI:** 10.3390/e27070770

**Published:** 2025-07-20

**Authors:** Beatrice Cairo, Riccardo Pernice, Nikola N. Radovanović, Luca Faes, Alberto Porta, Mirjana M. Platiša

**Affiliations:** 1 Department of Biomedical Sciences for Health, University of Milan, 20133 Milan, Italy; beatrice.cairo@unimi.it (B.C.); alberto.porta@unimi.it (A.P.); 2 Department of Engineering, University of Palermo, Viale delle Scienze, 90128 Palermo, Italy; luca.faes@unipa.it; 3 Pacemaker Center, University Clinical Center of Serbia, Faculty of Medicine, University of Belgrade, 11000 Belgrade, Serbia; nikolar86@gmail.com; 4 Faculty of Technical Sciences, University of Novi Sad, 21102 Novi Sad, Serbia; 5 Department of Cardiothoracic, Vascular Anesthesia and Intensive Care, IRCCS Policlinico San Donato, 20097 San Donato Milanese, Italy; 6 Laboratory for Biosignals, Institute of Biophysics, Faculty of Medicine, University of Belgrade, 11000 Belgrade, Serbia

**Keywords:** cardiorespiratory coupling, heart failure, information theory, cross-entropy, cross-predictability, k-nearest neighbors

## Abstract

The complex interplay between the cardiac and the respiratory systems, termed cardiorespiratory coupling (CRC), is a bidirectional phenomenon that can be affected by pathologies such as heart failure (HF). In the present work, the potential changes in strength of directional CRC were assessed in HF patients classified according to their cardiac rhythm via two measures of coupling based on k-nearest neighbor (KNN) estimation approaches, cross-entropy (CrossEn) and state space correspondence (SSC), applied on the heart period (HP) and respiratory (RESP) variability series, while also accounting for the complexity of the cardiac and respiratory rhythms. We tested the measures on 25 HF patients with sinus rhythm (SR, age: 58.9 ± 9.7 years; 23 males) and 41 HF patients with ventricular arrhythmia (VA, age 62.2 ± 11.0 years; 30 males). A predominant directionality of interaction from the cardiac to the respiratory rhythm was observed in both cohorts and using both methodologies, with similar statistical power, while a lower complexity for the RESP series compared to HP series was observed in the SR cohort. We conclude that CrossEn and SSC can be considered strictly related to each other when using a KNN technique for the estimation of the cross-predictability markers.

## 1. Introduction

Heart period (HP) is well known to be affected by respiratory phase, according to a mechanism termed respiratory sinus arrhythmia (RSA) [1]. While RSA is the best known aspect of cardiorespiratory interaction, the link between HP and respiratory activity encompasses multiple independent phenomena termed cardiorespiratory coupling (CRC) [2], and as such, different methodologies of both causal and non-causal nature have been proposed for its evaluation [3]. In particular, several studies [4,5,6,7,8,9,10,11,12,13] have explored both directions of cardiorespiratory interaction, namely evaluating the directionality from HP to respiration, and vice versa. Among them, measures such as cross-entropy (CrossEn) [14] and state space correspondence (SSC) methods [15] have been applied for the assessment of cardio-respiratory cross-predictability in both health [16] and pathology [7]. Both methodologies can be employed through the implementation of model-free k-nearest neighbor (KNN) approaches, making them comparable to one another.

Heart failure (HF) is a disease which has been assessed in the context of CRC through both unidirectional and bidirectional markers based on linear and nonlinear measures [6,7,17,18,19]. The results of these studies are valuable for a better understanding of the regulatory and compensatory mechanisms that include the autonomic nervous system and the baroreflex response, as a markedly increased sympathetic activity is present in HF. It is particularly challenging to examine the complexity of cardiac and respiratory signals, as well as their interactions in HF patients with arrhythmias, especially ventricular extrasystoles. In healthy individuals, ventricular premature contractions first cause, via the baroreceptor reflex, a drop in arterial pressure with an initial acceleration of the heart rate, followed by an increase in pressure and a decrease in the heart rate. This phenomenon is known as heart rate turbulence, and it is responsible for heart rate irregularity in healthy subjects [20]. This biphasic reaction induced by a premature ventricular ectopic beat is reduced or even completely eliminated in HF. Some studies have even shown that HF patients with ventricular extrasystoles have a more regular heart rate compared to those without premature beats [6]. What is certain is that CRC in the presence of ventricular extrasystoles has a strong nonlinear component and that the frequency of extrasystoles significantly influences it [7].

The autonomic nervous system (ANS) plays a decisive role in the provocation and maintenance of ventricular arrhythmia (VA) and sympathetic activation may provide both the trigger and substrate of VAs. This interaction between the ANS and VAs is expected in HF, which is a disease with a pronounced ANS imbalance, but has also been shown in the case of idiopathic extrasystoles, such as those from the outflow tract. Namely, it has been proven that patients with frequent outflow tract extrasystoles have impaired heart rate variability (HRV), in terms of significantly reduced values of HRV parameters in the time and frequency domains [21].

In the present work, we evaluate CRC in terms of the changes in the directional interaction of HP and respiratory signal dynamics using two methodologies based on KNN approaches (i.e., CrossEn and SSC), in order to investigate potential differences in either direction of interaction between HF patients with sinus rhythm (SR) and with VA, while accounting for the complexity of the cardiac and respiratory rhythms. To the authors’ knowledge, this study is the first comparison between the two methodologies in the context of CRC of HF patients, by using an extended version of the database in [6,7,17], classified according to their cardiac rhythm.

## 2. Materials and Methods

### 2.1. Subjects and Experimental Protocol

The data analyzed in this study were collected from patients with HF (reduced left ventricular ejection fraction, LVEF <35%) and indication for implantation of a cardiac resynchronization therapy (CRT) device or implantable cardioverter defibrillator (ICD), a subgroup of a database published in our previous works [7,17]. The group of patients with atrial fibrillation from this database was excluded from further analysis because heart rhythm in atrial fibrillation did not satisfy the basic condition of stationarity [22] for the methods of data analysis applied in the present work. The study was approved by the Ethics Committee of the Faculty of Medicine, University of Belgrade, and each subject signed an informed consent form (Approve Date: 17 March, Ref Numb 29/III-4). In this work, 25 HF patients with SR (age: 58.9 ± 9.7 years; 23 males) and 41 HF patients with VA (age 62 ± 11 years; 30 males) were considered. The VA group is defined according to the heart rhythm, i.e., with at least one ventricular extrasystole.

**Figure 1:**
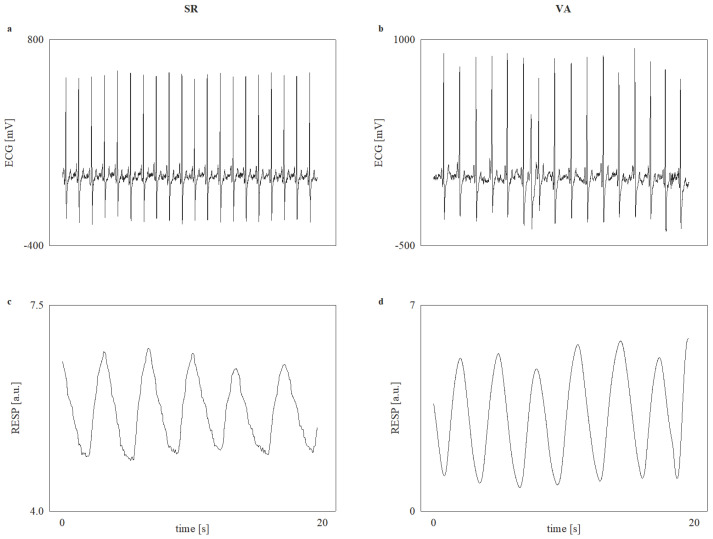
Examples of (**a**) the recorded ECG in one SR patient; (**b**) the recorded ECG in one VA patient; (**c**) the recorded RESP in the same SR patient; and (**d**) the recorded RESP in the same VA patient over an arbitrary period of 20 s. One isolated ventricular extrasystole can be observed in the ECG of the VA patient.

Experiments were conducted in the morning, between 7 and 8 a.m., in quiet surroundings in a room at the Pacemaker Center of the University Clinical Center of Serbia immediately before device implantation. The electrocardiogram (ECG) and the respiratory signal were acquired during 20 min of recording in relaxed subjects in the supine position and spontaneous breathing frequency without verbalization. Measurements were performed by using the Biopac MP100 system and Acqknowledge 3.9.1 software (BIOPAC System, Inc., Santa Barbara, CA, USA) using a 1 kHz sample rate and a 16-bit amplitude resolution. ECG data were acquired from lead I using the ECG 100C amplifier module, while an RSP 100C respiratory pneumogram amplifier module with TSD 201 strain gauge transducer attached to the belt was used to measure abdominal expansion and contraction as an estimation of the respiratory signal (RESP) [7,17]. Examples of the recorded signals are reported in Figure 1 for representative SR and VA subjects.

### 2.2. Series Extraction

R-wave peaks were identified on the ECG using a threshold-based algorithm applied to the first derivative of the ECG, and fixed by parabolic interpolation. Each HP value was estimated as the temporal interval between two consecutively identified R-wave peaks in order to compute the beat-to-beat HP series. A single sequence of 256 consecutive HP values of the beat-to-beat series were randomly selected for analysis for each patient and all identified fiducial points within the chosen window, namely the R-wave peaks, were visually checked. If erroneous detections were present, they were manually corrected using a strict criterion of a maximum of 5% of corrections; otherwise, the segment was discarded and a new one was selected. We did not analyze HP segments corresponding to periods of sustained arrhythmia even in the VA group to better compare similar sinus rhythm regimes in both cohorts and to focus on the chronic effects of arrhythmia on CRC in HF patients. Therefore, if isolated ectopic beats were visually identified, the series was linearly interpolated from the HP values involving the two beats most adjacent to the ectopic beat and classified as sinus rhythm. If any runs of consecutive ectopic beats were identified (i.e., transient arrhythmia), the segment was avoided and a different sequence of 256 HP values was considered. RESP was sampled in correspondence with the identified R-wave peaks in order to obtain a simultaneous beat-to-beat series. Within the beat-to-beat RESP series, the 256 consecutive values corresponding to the previously selected HP series segment were considered. Since the considered methodologies require stationarity, the fulfillment of this prerequisite was verified according to the stability of the mean and variance [22].

### 2.3. Cross-Entropy

Let us consider a bivariate random system **S** composed of two interacting processes *X* and *Y*. Given *n* as the present time, we denote as $X_{n}$ and $Y_{n}$ the univariate variables describing the present of the processes *X* and *Y*, while $X_{n}^{-}=\left[X_{n-1},\cdots ,X_{n-l}\right]$ and $Y_{n}^{-}=\left[Y_{n-1},\cdots ,Y_{n-l}\right]$ represent the vectors (of length *l*) describing their past.

The information-theoretic measure of CrossEn quantifies the amount of information carried by the present of the target that can be predicted solely by the past of the driver [23]:

(1) $$CrossEn_{X\to Y}=I\left(Y_{n};X_{n}^{-}\right)=H\left(Y_{n}\right)-H\left(Y_{n}|X_{n}^{-}\right)$$

with H(·) being the entropy of the present state of the process, H(·|·) the conditional entropy, and I(·;·) the mutual information between the processes [23]. According to this formulation, we neglect the instantaneous effects, i.e., the influence of the current state of the driver on the target process.

Herein, a non-linear model-free approach relying on *k*-nearest neighbors to compute the probability density functions has been exploited to compute the measure. The Kraskov–Stögbauer–Grassberger formulation has been employed to limit the bias arising from the combination of entropy terms evaluated on spaces of different dimensions [24]. According to this approach, the research of the *k*-nearest neighbors is performed in the highest-dimensional space, while the computation of the entropies is performed in the spaces of lower dimension through a distance-projection strategy. The CrossEn from *X* to *Y* can thus be estimated as follows:

(2) $$CrossEn_{X\to Y}=\psi \left(k\right)+\psi \left(N\right)-\langle \psi \left(N_{Y_{n}}+1\right)-\psi \left(N_{X_{n}^{l}}+1\right)\rangle$$

with *N* being the number of available realizations, ψ the digamma function, *k* the number of neighbors considered in the highest dimensional space, $N_{X_{n}^{l}}$ and $N_{Y_{n}}$ the numbers of patterns whose distances from the projected $x_{n}^{l}$ and $y_{n}$, respectively, are lower than the distance from the point $\left(x_{n}^{l},y_{n}\right)$ to its *k*-th neighbor. Further details on the derivation can be found in [25]. The obtained $CrossEn_{X\to Y}$ is thus an estimator of the X→Y directional interaction, higher values of which indicate a stronger interaction from *X* to *Y*.

In our analyses, we separately took into account both directions of CRC by first setting X as HP and Y as RESP, in order to evaluate the HP→RESP direction of interaction, and then X as RESP and Y as HP, in order to evaluate the opposite RESP→HP direction. If $CrossEn_{RESP\to HP}>CrossEn_{HP\to RESP}$, we considered RESP→HP as the dominant direction of interaction, and vice versa. In the present study, we set *k* to 20 and *l* to 2, corresponding to the parameters set for the SSC method described in Section 2.4. The analyses were carried out using MATLAB R2024b (MathWorks, Natick, MA, USA).

### 2.4. State Space Correspondence

Using the same notation as in Section 2.3 and in accordance with Takens’ theorem [26], $X^{-}=\left\{X_{n}^{-},l\leq \;n\leq N\right\}$ and $Y^{-}=\left\{Y_{n}^{-},l\leq \;n\leq N\right\}$ reconstruct the dynamic behavior of *X* and *Y* separately in the *l*-dimensional space, via delay coordinate embedding. In particular, the SSC method class hypothesizes the existence of a smooth function f(·) representing the link from *X*, taken as a driver, to *Y*, taken as a target. This is performed by mapping a point in the reconstructed phase space of *X* onto a point in the reconstructed phase space of *Y*. Specifically, different metrics have been proposed to verify the existence of this function and therefore of the link from *X* to *Y* within the umbrella of SSC approaches. Among them, cross-predictability (CP) [15] has been exploited in numerous physiological studies [15,16,27,28,29]. Specifically, we define the link from *X* to *Y* by evaluating the prediction of the current state of *Y* based on the past states of *X* [29,30], an approach recently demonstrated for stochastic systems with weak to moderate interactions such as those of CRC [16].

Specifically, we consider $X_{n}^{-}$ as the reference vector and $Y_{n}$ as the image of $X_{n}^{-}$. The *k*-nearest neighbors of $X_{n}^{-}$ in the embedding space of $X^{-}$ are used to compute the weighted mean of their images as a prediction of $Y_{n}$, hereafter represented as ${\hat{Y}}_{n}$, where the weights are the natural exponential of the inverse of their distance from $X_{n}^{-}$ [31].

The cross-predictability function (CPF) is defined over the embedding dimension *l* and is equal to the square correlation coefficient $\rho^{2}$ between $Y_{n}$ and ${\hat{Y}}_{n}$. The maximum over *l* of CPF was taken as a measure of the strength of the predictability of Y based on X, i.e., the link from *X* to *Y* [32], and referred to as the cross-predictability index (CPI) from *X* to *Y*. CPI is bound between 0 and 1, where null CPI indicates complete unpredictability of *Y* based on *X* and a null relationship from *X* to *Y*, while values near 1 indicate stronger prediction ability of the behavior of *Y* based on *X*, therefore a stronger relationship from *X* to *Y*.

In the present study, we set *k* to 20, in agreement with the approach used in [16]. We explored both directions of CRC by setting *X* and *Y* first as HP and RESP, respectively, in order to evaluate the HP→RESP direction of interaction, and then as RESP and HP, respectively, in order to evaluate the opposite RESP→HP direction. If $CPI_{RESP\to HP}>CPI_{HP\to RESP}$, we considered RESP→HP as the dominant direction of interaction, and vice versa [16].

### 2.5. Univariate Complexity Indexes

The complexity of the two considered series was assessed in order to evaluate the potential bias introduced by changes in the regularity of the driver and the target to the described interaction measures (see Section 2.3 and Section 2.4).

To evaluate the regularity and predictability of the time series, we first used the information storage (IS) measure, i.e., the quantity of information held in the current state of the system attributable to its past states, which is defined for a given process *X* as follows:

(3) $$IS_{X}=I\left(X_{n};X_{n}^{-}\right)=H\left(X_{n}\right)-H\left(X_{n}|X_{n}^{-}\right)$$

with H(·) being the entropy of the present state of the process, H(·|·) the conditional entropy, and I(·;·) the mutual information between the present and the past states of the process [23]. Using the non-linear KNN approach employing the Kraskov–Stögbauer–Grassberger formulation, IS for a given process *X* can be estimated as follows [24,33]:

(4) $$IS_{X}=\psi \left(k\right)+\psi \left(N\right)-\langle \psi \left(N_{x_{n}}\right)-\psi \left(N_{X_{n}^{l}}\right)\rangle$$

with *N* being the number of available realizations, ψ the digamma function, *k* the number of neighbors considered in the highest dimensional space, $N_{X_{n}^{l}}$ and $N_{x_{n}}$ the numbers of patterns whose distances from the projected $x_{n}^{l}$ and $x_{n}$, respectively, are lower than the distance from the point $\left(x_{n}^{l},x_{n}\right)$ to its *k*-th neighbor. In the present study, we set *k* to 20 and *l* to 2, corresponding to the parameters set for the CrossEn described in Section 2.3. The IS was computed separately for both HP and RESP by setting X=HP and X=RESP, respectively. Higher values of $IS_{HP}$ or $IS_{RESP}$ indicate lower complexity of the HP or RESP series, respectively.

Regarding the SSC approach, the degree of predictability of $X_{n}$ based on $X_{n}^{-}$ was used to compute a predictability function (PF) bound between 0 and 1, in a procedure completely analogous to that described in Section 2.4 where the reference vector was set to $X_{n}^{-}$. A predictability index (PI) was then computed as the maximum of PF over *l*, and taken as an index of complexity. In the present study, we set *k* to 20, corresponding to the parameters set for CPI in Section 2.4. PI was computed separately for both the HP and RESP series by setting X=HP and X=RESP, respectively [34]. Higher values of $PI_{HP}$ or $PI_{RESP}$ indicate lower complexity of the HP or RESP series, respectively.

### 2.6. Statistical Analysis

The normality of the distributions was verified via the Shapiro–Wilk test. For all clinical variables, t-tests, or Mann–Whitney U tests when appropriate, were carried out over the continuous variables, while chi-square tests, or Fisher Exact tests when appropriate, were applied for the categorical variables in order to evaluate the differences between the two experimental groups (i.e., SR and VA). For the directional CRC indexes, a two-way repeated measure ANOVA with one factor repetition was performed to evaluate the differences between the directions of interaction (i.e., HP→RESP and RESP→HP) within the same experimental group and between cohorts (i.e., SR and VA) within the same direction of interaction. Multiple comparisons were performed post hoc using the Holm–Sidak method.

A mixed model analysis was performed to account for the potential effect of the clinical confounding factors on each index of cardiorespiratory interaction (i.e., CrossEn and CPI). Specifically, a linear mixed model for repeated measures was employed, using the experimental group and direction of interaction as fixed effects and the clinical variables that showed a statistically significant difference among the two cohorts as the random effect.

Statistical analyses were performed using commercial statistical software (Sigmaplot v.14.0, Systat Software, San Jose, CA, USA and IBM SPSS Statistics v.22.0.0.0, Armonk, NY, USA: IBM Corp.). A *p* < 0.05 was always deemed significant.

## 3. Results

Table 1 represents the clinical characteristics of the two cohorts (i.e., SR and VA). No statistically significant differences can be observed between SR and VA in terms of age, gender or etiology, while it can be observed that a significantly higher percentage of VA patients had been diagnosed with HF for more than two years, presented a significant number of VES in the 24 h ECG Holter recording performed before the experiment, and had a history of hypertension. No other significant differences were observed in both comorbidities and clinical indicators of cardiac function, such as the LVEF and NYHA class.

**Figure 2:**
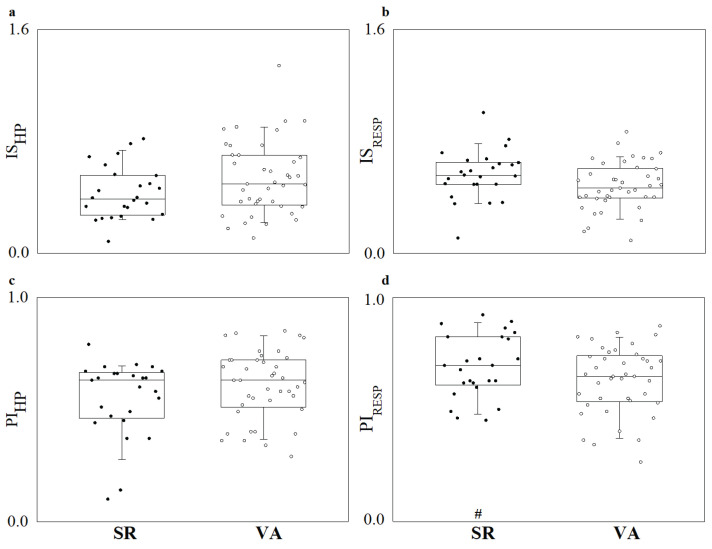
Boxplots reporting (**a**) information storage (IS) for the HP series; (**b**) IS for the RESP series; (**c**) predictability index (PI) for the HP series; and (**d**) PI for the RESP series. # indicates significant difference between target variables (i.e., HP vs. RESP).

Figure 2 reports the complexity indexes of IS (panels a and b) and PI (panels c and d) as boxplots with the individual values for each patient for the evaluation of the regularity of HP (panels a and c) and RESP (panels b and d). It can be observed that IS (panels a and b) and PI (panels c and d) show a non-significant change for both the HP (panels a and c) and RESP (panels b and d) series when considering VA patients compared to SR patients. Furthermore, only SR patients show a significantly higher predictability for the RESP series compared to the HP series when considering the PI index, while IS shows a similar trend but no statistical significance.

**Figure 3:**
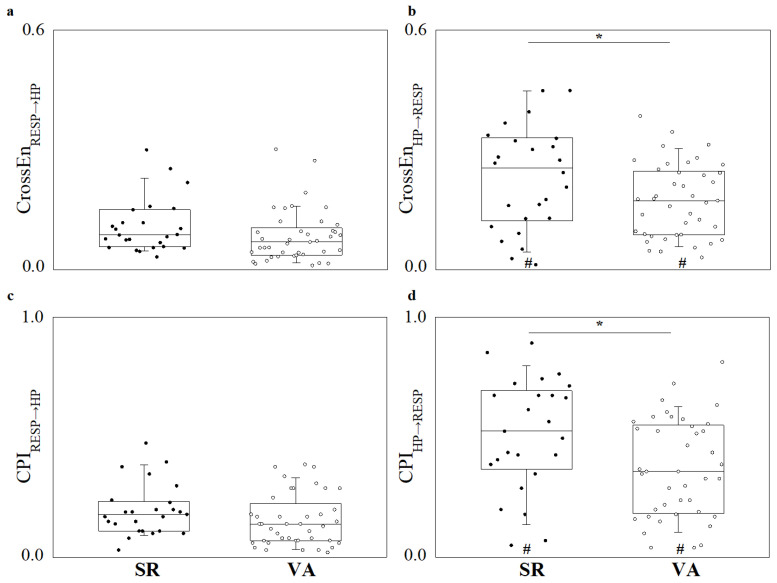
Boxplots reporting (**a**) cross-entropy (CrossEn) in the RESP→HP direction; (**b**) CrossEn in the HP→RESP direction; (**c**) cross-predictability index (CPI) in the RESP→HP direction; and (**d**) CPI in the HP→RESP direction. * indicates significant difference between experimental groups (i.e., SR vs. VA); # indicates significant difference between directions of interaction (i.e., RESP→HP vs. HP→RESP).

Figure 3 reports the CRC indexes of CrossEn (panels a and b) and CPI (c and d) as boxplots with the individual values for each patient for the evaluation of the directional interaction RESP→HP (panels a and c) and HP→RESP (panels b and d). It can be observed that neither index (CrossEn, panel a; CPI panel c) shows a significant change in the RESP→HP interaction between experimental cohorts, while a significant decrease in both CrossEn (panel b) and CPI (panel d) can be observed in the HP→RESP direction when considering VA patients compared to SR patients. Both CrossEn and CPI also show significantly higher values when considering the direction of interaction HP→RESP when compared to RESP→HP in both SR and VA cohorts.

Regarding the linear mixed model analysis, we observed that for all CRC indexes, the random effect of clinical variability within cohorts never reached statistical significance (*p* > 0.05) and further results are therefore not reported.

## 4. Discussion

### 4.1. Clinical Characteristics

The differences in the clinical characteristics of the two groups of HF patients were largely expected. Although ventricular extrasystoles can be completely benign in nature, they can also be an excellent indicator of a serious disorder. Especially when extrasystoles are multifocal and prone to clustering, electrolyte disturbances, structural heart disease, or coronary artery disease should be considered. Our results showed that HF patients with VA more often had ischemic heart disease (although this difference was not statistically significant), and were treated significantly longer for HF. In terms of comorbidities and risk factors for the development of cardiovascular disease, the VA patients presented a higher incidence of hypertension, but there was a relative homogeneity for other risk factors, excluding them as a confounding factor for our further results, as confirmed by the linear mixed model analysis. An important finding is that most of these patients had a lower NYHA class, as malignant ventricular arrhythmias, i.e., sudden cardiac death, have been shown to be a greater problem in patients who are not in advanced/terminal HF [35].

### 4.2. Complexity of Heart and Respiratory Rhythms

While some previous studies have found that the occurrence of ventricular extrasystoles in HF further reduces the irregularity, or complexity, of the heart rhythm, when measured by sample entropy, our results indicate that the cardiac rhythm complexity is not significantly different in the VA group, with a slight but not statistically significant increase in regularity compared to the SR cohort [6]. The complexity of HP in these patients is definitely influenced by multiple factors. It is not only important whether ventricular extrasystoles occur, but also how frequent they are. Furthermore, the severity of HF, or the degree of parasympathetic reduction, is also important, and we must always keep in mind that most drugs used in the treatment of HF actively affect the tone of the sympathetic and parasympathetic system [36,37].

Respiratory signals consist of oscillatory and deterministic characteristics, but it was unexpectedly discovered, by the results of the respiratory signal analysis, that the complexity of RESP usually was similar to the complexity of the HP time series [38,39]. This finding challenged the prevailing assumption that HP would inherently exhibit greater complexity due to the multifaceted control systems involved in cardiac regulation. This expected result was only confirmed in the SR cohort for the PI index, where an increased predictability of RESP can be observed, confirming results obtained on healthy subjects and athletes [16], but not when IS was applied on the same population, although similar trends are present, showing a different statistical power of the two methodologies. Researchers surmised that the neural and mechanical interactions governing respiratory patterns may contribute to this unexpected complexity in RESP.

Several factors taken together probably contribute to the findings related to the change in complexity of the respiratory rhythm of HF patients in SR, compared to the complexity of their heart rhythm, as observed via the PI marker. The fundamental mechanism underlying the neural control of breathing is a three-component system which ensures the proper regulation of respiratory patterns. The brainstem central pattern generator, particularly the preBötzinger complex with inhibitory neurons active during inspiratory bursts, plays a crucial role as the primary rhythm driver, generating the basic breathing pattern. The principal role of chemoreceptors in the control of breathing, in the central nervous system and peripheral nervous system, is as feedback detectors of errors in pH and partial pressures of CO_2_ and O_2_ in the arterial blood, and to a lesser extent to a tonic control of breathing. The third component comprises efferent outputs of the muscles of the respiratory pump and airways [40]. Breathing is a behavior that must exhibit plasticity defined as a persistent change in the neural control system, morphology, and/or function, based on experience [41]. Plasticity within the respiratory control network can be manifested at multiple levels: it may occur at any level of respiratory control, including carotid body chemoreceptors, brainstem central pattern generators, and/or spinal α-motor neurons [42]. As demonstrated by Ainsworth and colleagues (1992), vagal blockade in resting or exercising dogs prolongs inspiratory time, reduces breathing frequency, and augments tidal volume [43]. This indicates that vagal modulation plays a significant role in respiratory control, contributing to the complexity and adaptability of respiratory patterns. In patients with congestive HF, heightened chemoreflex sensitivity manifests as augmented sympathetic outflow, ventilatory instability, and augmented ventilatory responses to exercise [44]. Furthermore, this form of congestive HF-induced increase in carotid body plasticity is attributed to a shift in redox balance toward oxidative stress, resulting in endothelial dysfunction [45]. Marcus and colleagues (2018) further elucidate that this oxidative stress-mediated plasticity is a pivotal factor in the heightened chemoreflex sensitivity observed in HF patients. Their research indicates endothelial dysfunction as a significant contributor to altered respiratory patterns and autonomic dysregulation in HF [46]. Radaelli et al. showed that in free-breathing HF patients, compared with control subjects, a greater modulation of respiratory volumes in the very-low frequency range is present, which contributes to preserving the baroreflex-mediated control of the heart rate [47].

While similar trends can be observed for both IS and PI of both HP and RESP, only PI found a higher predictability of RESP compared to HP, which was not observed with IS. Methodologically, the lower statistical power of IS can be related to the fact that this measure reflects the whole information contained in the past of the target that can be used to predict its present, no matter of the origin of such information. Therefore, IS does not strictly reflect the internal dynamics in the target system but is influenced as well by the causal interactions from source to target [23].

### 4.3. Cardiorespiratory Coupling

In the present study, we considered the intricate interplay between the cardiac and respiratory systems in the context of HF, and it is apparent that complementary measures of cardiorespiratory coupling highlighted a different influence of the heart rhythm on respiration between patients’ groups. In the group of HF patients with SR, we observed a stronger influence of the heart rhythm on the respiratory rhythm, as evaluated by CrossEn and CPI, than in the other group. We may hypothesize that, in HF patients with VA, some respiratory plasticity-related mechanisms are lost compared to SR, which in return decreases the heart rhythm influence on respiratory patterns. This result was obtained in the absence of any change in PI across experimental groups and only PI of RESP was found to be higher than PI of HP for the SR cohort. It must be taken into account that this last result might introduce a bias in the calculated CRC markers when considering the RESP→HP direction, as it is known from simulation studies [16] that an increased complexity of the target compared to that of the driver influences the measures considered in the present study. Therefore, in the RESP→HP direction, the bias introduced by the observed change in complexity for the SR cohort might mask the presence of a significant difference between cohorts on the CRC markers in this direction as well. On the other hand, the results in the HP→RESP are not as influenced by this result as the driver is more complex than the target and therefore the significant difference between SR and VA is confirmed.

Furthermore, this confirms previous results [7] investigating the two temporal directions of CRC on HF patients, finding a stronger interaction in the HP→RESP direction than in the opposite one, which is more commonly investigated. This direction of interaction, termed cardioventilatory coupling [3,5,16,48,49], seems to be dominant in the present application. It has been hypothesized [4] that the inspiratory onset might be triggered by an event associated with the preceding heartbeat, although the cardiovascular afferent mechanisms that might be responsible for initiating these reflexes are not yet known. Regardless, these mechanisms seem to be more compromised in HF patients with VA, although the level at which regulatory systems cause this phenomenon to occur in the context of VA remains unclear [7]. This result stresses the importance of investigating both arms of the closed loop interaction between HP and RESP for a complete assessment of CRC in health and pathology.

Of interest, results of bidirectional CRC obtained with the two methodologies yielded the same trends and statistical significance, proving the strong correlation between the two measures, which can be attributed to both methods implementing the concept of cross-predictability with a similar estimation approach. The CrossEn measure represents the cross-information from the driver to the target process, i.e., the overall amount of information carried by the present of the target that can be explained by the past of the driver, including not only the causal interaction from the driver to the target, but also the causal interactions from the target to the driver, and the contribution from the contemporaneous presence of internal dynamics in the target [23], while CPI is grounded on the reconstruction of the current state of the target based on the past states of the driver, by testing the existence of a functional relationship between the two.

Usually, HF patients exhibit both diminished variability in heart rate [50] and a reduction in the flexibility of cardiorespiratory coupling [6,18], underscoring the importance of alternative adaptive mechanisms when global autonomic input is compromised. In such scenarios—where parasympathetic tone is blunted and cardiac performance is compromised—the analytical framework employed in this study becomes particularly valuable. By quantifying the directionality and relative potency of cardiorespiratory interactions, these methods can reveal subtle shifts in physiological regulation that traditional metrics might overlook. The ability to disentangle the residual, non-vagal influences on heart–respiratory coupling offers a window into compensatory pathways that may sustain function in advanced disease. This nuanced perspective provides clinicians and scientists with more precise tools to assess the adaptive landscape of the failing heart, informing both prognosis and potential targets for intervention. This interpretation is substantiated by our results indicating a reduced influence from HP signals to respiratory activity within the VA cohort. The underlying pathophysiology of VA frequently stems from structural and functional alterations in the ventricular myocardium, which disrupt the usual feedback mechanisms from cardiac to respiratory centers. As a result, the capacity of the heart to modulate respiratory rhythms is attenuated, reflecting a fundamental shift in the hierarchy and reciprocity of cardiorespiratory control. Such findings highlight the intricate dependence of bidirectional physiological regulation on cardiac structural integrity, especially when traditional autonomic pathways are compromised.

Clinical practice shows that in patients with HF, especially those with reduced LVEF, there are no sufficiently good predictors or composite risk scores to identify patients with an increased risk of sudden cardiac death or those who will benefit from a certain therapeutic option, such as vagus nerve stimulation or MitraClip implantation, or cardiac resynchronization therapy [51]. Predictors are mainly sought among the parameters obtained by applying modern imaging techniques that assess the presence of anatomic substrate abnormalities, then among ECG-derived parameters, or the presence of various comorbidities [52]. In a previous study [17], we identified the long-term scaling exponent of the RR interval series as the parameter that could preoperatively separate future responders from those who would not benefit from resynchronization therapy. We believe that the measures used in this work, assessing the cardiac and respiratory rhythm complexity, and primarily the coupling of these two signals, will be valuable in future studies in addressing some of the numerous open clinical questions.

### 4.4. Limitations and Future Developments

In this study, we did not analyze RESP segments corresponding to HP segments with arrhythmias, even in the VA cohort, in order to better compare similar regimes of sinus rhythm in the two cohorts and focus on the chronic effects of arrhythmia on CRC and the complexity of the variability series in HF. Hence, we did not look at the effect of the temporary appearance of VAs in HF patients on the respiratory rhythm and CRC. In the future, the acute effect of arrhythmic epochs and/or atrial fibrillation on CRC in HF should be studied.

Furthermore, in this exploratory study, the considered techniques of CRC assessment were tested in offline and controlled laboratory conditions. In the future, their validation for real-time clinical use should be performed, aiming for integration into monitoring systems for the clinical assessment of CRC.

It must also be noted that the proposed measures of CrossEn and SSC are directional but not causal as defined by the Granger causality (GC) paradigm [53]. GC measures can be implemented using similar KNN techniques [54,55] and thus could be computed in future studies in parallel, with the present directional coupling measures to investigate the differences between the two approaches. Differently than measures of cross-predictability, GC approaches discount internal effects on the target system by computing the statistical dependence between the past of the driver series and the present of the target series after conditioning on the past of the target.

Finally, it is worth noting that one of the limitations of this study is that we did not involve the group of healthy control subjects who were closer in age to the HF patients. The demographic realities of aging populations, coupled with the high prevalence of subclinical cardiovascular risk factors, render the identification of truly healthy elderly individuals exceptionally rare. Despite this limitation, the present study offers a robust framework for interrogating HF disease-specific alterations in cardiorespiratory dynamics while highlighting the pressing need for innovative approaches to control group selection in translational cardiovascular research.

Future studies could focus on the context of heart transplant recipients, whose donor hearts are entirely vagally denervated and consequently lack RSA. In such patients, the absence of vagal influence effectively isolates non-autonomic, intrinsic mechanisms of cardiorespiratory coupling. This unique physiological condition would provide an unambiguous substrate for evaluating the full clinical relevance of our analytical approach. Additionally, quantifying changes in directionality and strength of cardiorespiratory interactions could help differentiate between adaptive and maladaptive responses in individuals with varying degrees of cardiorespiratory compromise. For instance, the restoration or loss of specific feedback loops during apneas, as opposed to normative sleep-breathing cycles, may serve as functional markers for disease progression or therapeutic efficacy. Such analyses might further illuminate how the interplay between parasympathetic withdrawal, sympathetic surges, and altered baroreflex sensitivity modulates the overall cardiorespiratory landscape in both acute and chronic settings.

## 5. Conclusions

Cross-predictability methodologies such as cross-entropy and SSC approaches revealed changes in CRC between HF patients with SR and VA, under the hypothesis of loss of respiratory plasticity-related mechanisms in HF patients with VA, compared to HF patients with SR. Moreover, we observed a predominant directionality of interaction from the cardiac rhythm to the respiratory rhythm in both cohorts and using both methodologies, with similar statistical power. We conclude that the two studied approaches can be considered strictly related to each other when using a KNN technique for the estimation of the CrossEn and CPI markers. Furthermore, we stress the importance of considering directionality of interaction by applying causal markers in the assessment of CRC, and of accounting for the complexity of the target in the evaluation of the driver to target relationship, for a more complete evaluation of the dynamics of two weakly coupled systems such as the heart and respiratory system.

## Figures and Tables

**Table 1:** Clinical characteristics of HF patients with sinus rhythm (SR) and ventricular arrhythmia (VA).

Clinical Variable	SR (N = 25)	VA (N = 41)
Age [years]	58.9 ± 9.7	62.2 ± 11.0
Gender [male]	23 (92)	30 (73)
Ischemic etiology	12 (48)	27 (66)
Disease duration < 2 years	18 (72)	13 (32) *
History of paroxysmal atrial fibrillation	3 (12)	7 (17)
Significant number of VES on the 24 h ECG Holter before recording	6 (24)	23 (56) *
LVEF [%]	25.8 ± 5.6	25.3 ± 5.8
NYHA class II	20 (80)	34 (83)
NYHA class III	5 (20)	7 (17)
Hypertension	11 (44)	33 (80) *
Diabetes mellitus	5 (20)	13 (32)
Dyslipidemia	13 (52)	26 (63)
Tobacco smoking	11 (44)	23 (56)
COPD	2 (8)	3 (7)

Categorical variables are presented as number (percentage). Continuous variables are presented as mean ± standard deviation. The symbol * indicates a significant difference with *p* < 0.05. N = number of patients for each group; VES = ventricular extrasystoles; LVEF = left ventricular ejection fraction; NYHA = New York Heart Association; COPD = chronic obstructive pulmonary disease.

## Data Availability

Data will be made available on request.

## Abbreviations

The following abbreviations are used in this manuscript:

HP	heart period
RSA	respiratory sinus arrhythmia
CRC	cardiorespiratory coupling
CrossEn	cross-entropy
SSC	state space correspondence
KNN	k-nearest neighbor
HF	heart failure
SR	sinus rhythm
VA	ventricular arrhythmia
LVEF	left ventricular ejection fraction
CRT	cardiac resynchronization therapy
ECG	electrocardiogram
RESP	respiration
CP	cross-predictability
CPF	cross-predictability function
CPI	cross-predictability index
IS	information storage
PF	predictability function
PI	predictability index
